# Complete chloroplast genome sequence of *Peganum harmala,* an important medicinal plant

**DOI:** 10.1080/23802359.2019.1711230

**Published:** 2020-01-16

**Authors:** Xi Zha, Pengyuan Zhao, Fei Gao, Yijun Zhou

**Affiliations:** College of Life and Environmental Sciences, Minzu University of China, Beijing, People’s Republic of China

**Keywords:** *Peganum harmala*, chloroplast genome, phylogenetic analysis

## Abstract

*Peganum harmala* is a perennial herbaceous plant belonging to the genus Peganum, Nitrariaceae and is mainly distributed in dry areas in the Mediterranean and many Asia countries. This plant species has high medicinal value and considerable ecological value. This article reports the first chloroplast genome of species in Peganum. The size of the *P. harmala* chloroplast genome is 160,070 bp, including a large single-copy (LSC) region (88,279 bp), a small single-copy (SSC) region (26,468 bp), and two reverse (IR) regions (18,856 bp). The *P. harmala* chloroplast genome consists of 132 genes, including 83 protein-coding genes, 38 transfer RNA (tRNA) genes, and 8 ribosomal RNA (rRNA) genes. The GC content of *P. harmala* chloroplast genome is 36.44%. Phylogenetic analysis showed that *P. harmala* and another Nitrariaceaeis species formed a single blade in the phylogenetic tree.

*Peganum harmala*, also called as Syrian rue or wild rue is a perennial herbaceous plant native to the Mediterranean and various regions of Asia. The plant is frequently used as folk medicine in Middle Eastern, North Africa, and China (Airaksinen and Kari 1981). Alkaloids extracted from *P. harmala* seeds have many pharmacological activities including bactericidal (Shaheen and Issa 2019), insecticidal, and anti-tumor effects (Lamchouri and Université Sidi Mohamed Ben Abdellah 2014), and especially, the harmala alkaloids harmine and harmaline, two highly psychoactive alkaloids, act as reversible monoamine oxidase inhibitors (Berrougui et al. 2012). In addition, *P. harmala* usually grows in dry and saline soils in the temperate desert and Mediterranean regions and thus is also an ecologically important plant species.

There are different opinions on the taxonomic status of *Peganum*. The genus *Peganum* was classified into the family Zygophyllaceae in Flora Reipublicae Popularis Sinicae (Xu and Huang 1998), into the Pelicaceae family in Flora of China (Liu and Zhou 2008), and into the Nitrariaceae in the APG IV system (Chase et al. 2016). The sequence and structure of the chloroplast genome are highly conserved, making it an ideal tool for studying plant evolution and phylogenetics (Cheng et al. 2019). In the present study, fresh leaves of *P. harmala* were collected from adult plants in Erdos City (39°49′51.48″N, 106°47′21.17″E), Inner Mongolia Autonomous Region, China. The leaf sample (20181001-02) was stored in the College of Life and Environmental Sciences, Minzu University of China, Beijing. Total genomic DNA was extracted from the leaves using Plant Genomic DNA Kits (Tiangen Biotech Co., Beijing, China) and sequenced on an Illumina Hiseq2500 platform. The sequencing data was assembled using spades v.3.13.1 (Bankevich et al. 2012) and annotated with PGA (Qu et al. 2019). The *P. harmala* chloroplast genome sequence was deposited in GenBank (Accession Number: MK341054).

The complete chloroplast genome of *Peganum harmala* was 160,070 bp in length and encode 132 genes, including 86 protein-coding genes, 38 tRNAs, and 8 ribosomal RNA genes. In chloroplast genome of *P. harmala*, *trnK-UUU*, *rps16*, *trnG-UCC*, *atpF*, *rpoC1*, *trnL-UAA*, *trnV-UAC*, *petB*, *petD*, *rpl16*, *rpl2*, *ndhB*, *trnI-GAU*, *trnA-UGC*, *and* *ndhA* genes contain one intron, and *clpP and ycf3* contain two introns.

To analyze the phylogenetic status of *P. harmala*, a total of 36 complete chloroplast genome sequences belonging to Sapindales were downloaded from RefSeq database and *Amborella trichopoda* was set as an outgroup. For the phylogenetic analysis, 51 common protein-coding sequences from the 38 species were aligned using MAFFT V7.450 (Katoh and Standley 2013), the alignment was manually examined and adjusted and a phylogenetic tree was constructed by MEGA X (Kumar et al. 2018) software using maximum likelihood method with 1000 bootstrap repeats (Figure 1). The results showed that *P. harmala* and *Nitraria tangutorum*, another Nitrariaceae species (Abla et al. 2019), formed a single blade in the phylogenetic tree. The Nitrariaceae blade is the basal branch among Nitrariaceae species, suggested that Nitrariaceae was differentiated from other Sapindales plants a long time ago. In brief, our study provides important data for understanding the phylogenetic status of plant species in Nitrariaceae.

**Figure 1.**
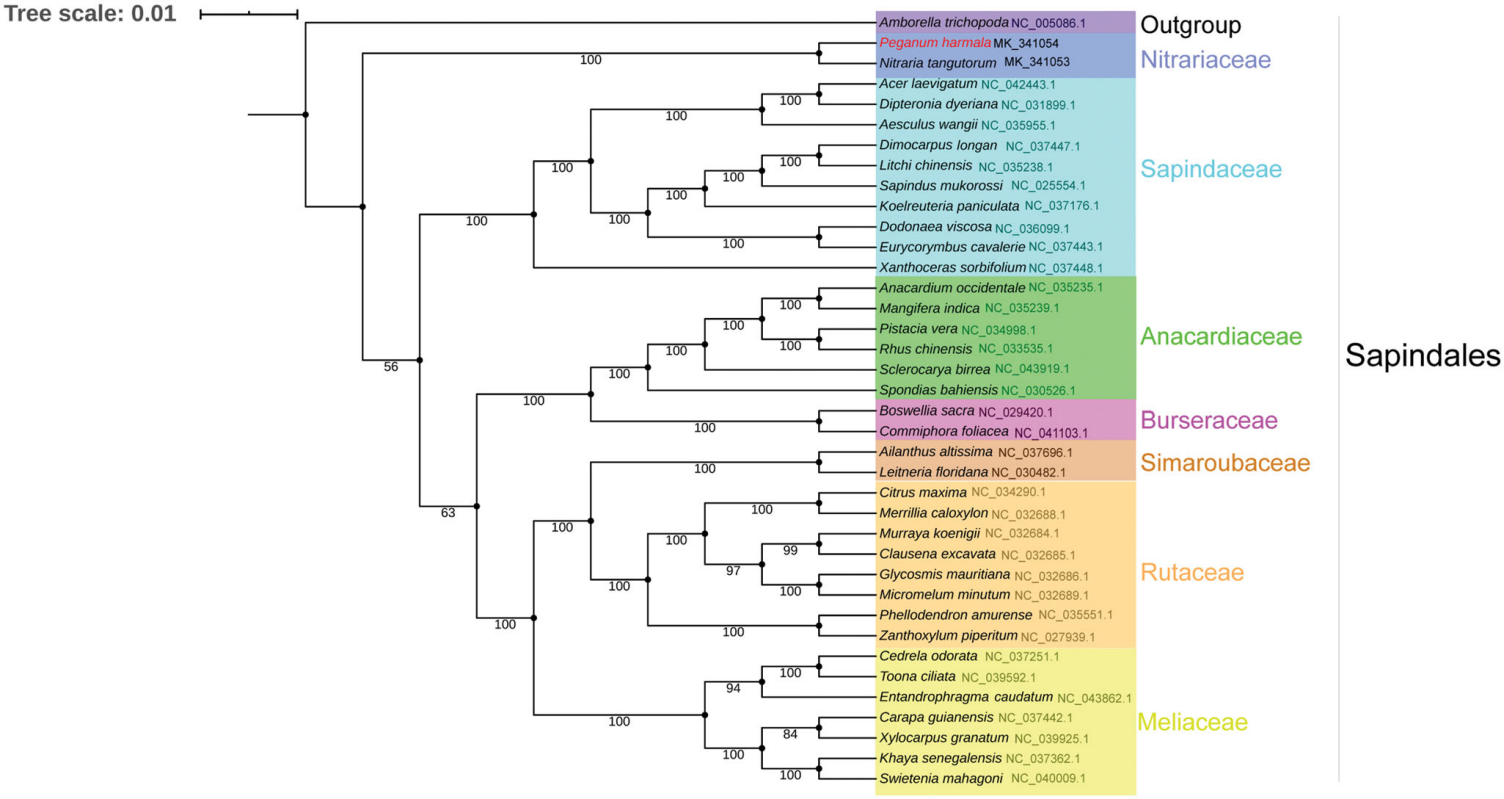
A phylogenetic tree constructed based on 38 complete chloroplast genome sequences. Bootstrap support is indicated for each branch. GenBank accession numbers: *Peganum harmala* (MK_341054), *Nitraria tangutorum* (MK_341053), *Amborella trichopoda* (NC_005086.1), *Acer laevigatum* (NC_042443.1), *Aesculus wangii* (NC_035955.1), *Ailanthus altissima* (NC_037696.1), *Anacardium occidentale* (NC_035235.1), *Boswellia sacra* (NC_029420.1), *Carapa guianensis* (NC_037442.1), *Cedrela odorata* (NC_037251.1), *Citrus maxima* (NC_034290.1), *Clausena excavate* (NC_032685.1), *Commiphora foliacea* (NC_041103.1), *Dimocarpus longan* (NC_037447.1), *Dipteronia dyeriana* (NC_031899.1), *Dodonaea viscosa* (NC_036099.1), *Entandrophragma caudatum* (NC_043862.1), *Eurycorymbus cavaleriei* (NC_037443.1), *Glycosmis mauritiana* (NC_032686.1), *Khaya senegalensis* (NC_037362.1), *Koelreuteria paniculata* (NC_037176.1), *Leitneria floridana* (NC_030482.1), *Litchi chinensis* (NC_035238.1), *Mangifera indica* (NC_035239.1), *Merrillia caloxylon* (NC_032688.1), *Micromelum minutum* (NC_032689.1), *Murraya koenigii* (NC_032684.1), *Phellodendron amurense* (NC_035551.1), *Pistacia vera* (NC_034998.1), *Rhus chinensis* (NC_033535.1), *Sapindus mukorossi* (NC_025554.1), *Sclerocarya birrea* (NC_043919.1), *Spondias bahiensis* (NC_030526.1), *Swietenia mahagoni* (NC_040009.1), *Toona ciliate* (NC_039592.1), *Xanthoceras sorbifolium* (NC_037448.1), *Xylocarpus granatum* (NC_039925.1), *Zanthoxylum piperitum* (NC_027939.1).
